# Enabling nutrient security and sustainability through systems research

**DOI:** 10.1007/s12263-015-0462-6

**Published:** 2015-04-16

**Authors:** Jim Kaput, Martin Kussmann, Yery Mendoza, Ronit Le Coutre, Karen Cooper, Anne Roulin

**Affiliations:** Nestlé Institute of Health Sciences, Lausanne, Switzerland; Nestlé Research Center, Lausanne, Switzerland; Regulatory and Scientific Affairs, Nestlé, Vevey, Switzerland; Nutrition, Health and Wellness and Sustainability, Nestlé, Vevey, Switzerland

**Keywords:** Nutrient sustainability, Nutrient security, Systems nutrition, Health, Nutrient chain

## Abstract

Human and companion animal health depends upon nutritional quality of foods. Seed varieties, seasonal and local growing conditions, transportation, food processing, and storage, and local food customs can influence the nutrient content of food. A new and intensive area of investigation is emerging that recognizes many factors in these agri-food systems that influence the maintenance of nutrient quality which is fundamental to ensure nutrient security for world populations. Modeling how these systems function requires data from different sectors including agricultural, environmental, social, and economic, but also must incorporate basic nutrition and other biomedical sciences. Improving the agri-food system through advances in pre- and post-harvest processing methods, biofortification, or fortifying processed foods will aid in targeting nutrition for populations and individuals. The challenge to maintain and improve nutrient quality is magnified by the need to produce food locally and globally in a sustainable and consumer-acceptable manner for current and future populations. An unmet requirement for assessing how to improve nutrient quality, however, is the basic knowledge of how to define health. That is, health cannot be maintained or improved by altering nutrient quality without an adequate definition of what health means for individuals and populations. Defining and measuring health therefore becomes a critical objective for basic nutritional and other biomedical sciences.

Sustainable nutrition: The physical and economic access to sufficient, safe and nutritious food and water to fulfill dietary and cultural needs to enable an active and healthy lifestyle…without compromising the ability of future generations to meet these needs (Espinoza-Orias et al. 2014).

## Global nutrition status today

**Fig. 1:**
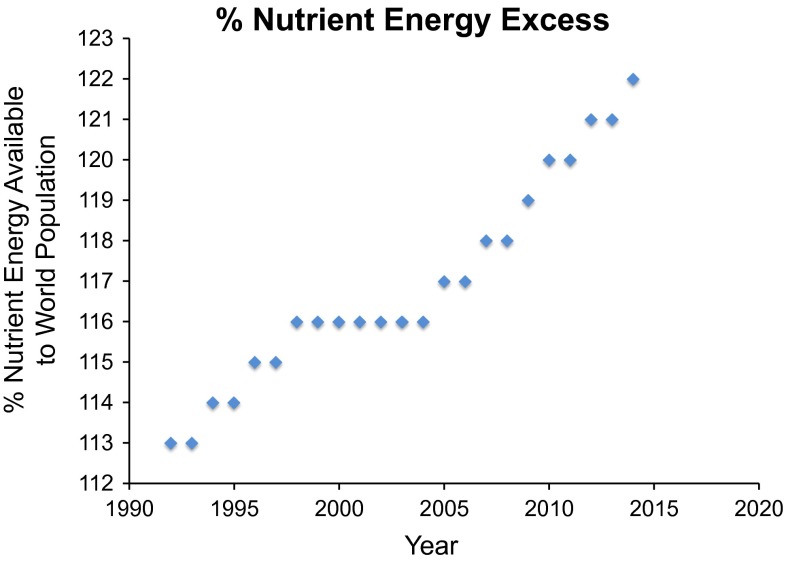
Projected nutrient excess to world populations 1990–2015. While populations in certain regions lack enough calories for growth and health, sufficient calories are produced to feed the world’s populations. Source: http://faostat3.fao.org/browse/D/FS/E

**Fig. 2:**
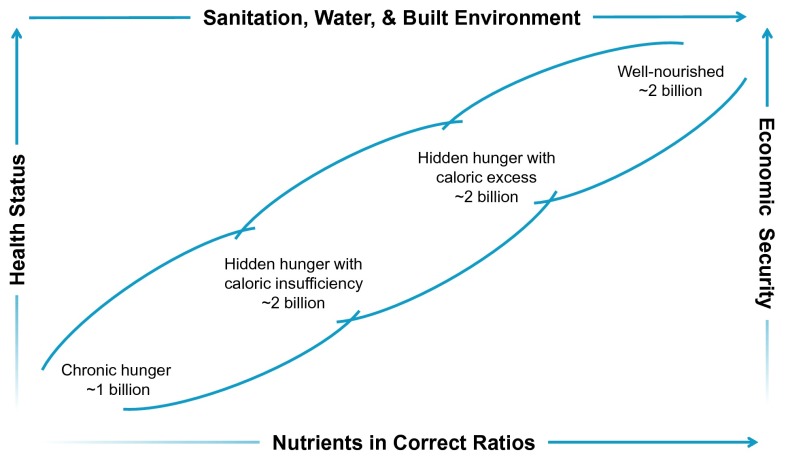
Continuum of health in world populations. Health results not only from nutrients in correct ratios, but also is influenced by economic security and the built environment with access to sanitation and clean water as most important. The *arrow* signifies these are continuous rather than discrete variables or phenotypic conditions

More calories are available today to a higher percentage of the world’s population than at any time in human history (Fig. 1). Yet, about 800 million people are undernourished resulting in wasting and stunting, 2 billion lack essential nutrients, and 2 billion suffer from over-nutrition resulting in excess weight or obesity. Micronutrient deficiencies and insufficiencies currently affect between 2 and 3 billion of the world’s population (Haddad et al. 2014) resulting in a reduced potential to attain full physical and cognitive development. Imbalanced micronutrient intake may also contribute to obesity and its related complications (Fig. 2). The uneven food and nutrient distribution across the planet caused by socioeconomic and political factors will be exacerbated by the growth of the world population, global climate change, increasing water scarcity and its limited accessibility, and diminishing agricultural land resources (Acharya et al. 2014). By 2050, the gap in average daily energy requirements of 2300 kcal per day will be between 200 and 950 kcal per person depending on the amount of food waste (Searchinger et al. 2013). The true nutrient insufficiencies may be greater since energy intake rather than nutrient density is used in these calculations.

While the biomedical community understandably focuses its research on human biology, providing the right nutrients to populations living in different environmental contexts requires an integrated (eco)system and “nutrient chain” view from soils via plants and animals to food and the consumer (Tilman and Clark 2014). Nutritional quality can only be assured if environmental sustainability is preserved and economic viability is maintained (Hammond and Dubé 2012; Acharya et al. 2014; Herforth et al. 2014). These interlocked domains are often viewed and discussed from high-income countries’ perspectives of large-scale agriculture and big business regulated by national and local governments, all of which are crucial for providing safe and affordable food. However, it is families that own and operate over 70 % of the 570 million farms, and it is the latter that produce more than 80 % of the value of the world’s food (FAO 2014). The 1 % of large-scale farms (larger than 50 ha) controlling 65 % of the world’s agricultural land are typically found in high- and middle-income countries. In contrast, 95 % of the farms in low-income and lower-middle income countries are smaller than 5 ha (FAO 2014). Maintaining or improving nutrient quality on farms in diverse environments with different access to water and other resources (e.g., fertilizers) will require targeted methods and approaches appropriate to the climate conditions, environmental contexts, cultural acceptance, and socioeconomic and educational circumstances of not only the mega-farms but also those of communities and families. The U.S. Institute of Medicine—National Research Council outlined a framework to assist in food and agriculture decision-making consisting of six steps that (1) identify the problem, (2) define the scope of assessment, (3) identify scenarios for new policies, (4) conduct the analysis, (5) synthesize the results, and (6) report the findings to appropriate stakeholders (Institute of Medicine 2015).

Food production and manufacturing are also considered to be dominated by global companies. However, in reality, food processing is predominantly small scale and local in nature. The market share of the top 50 food and beverage companies account for <20 % of global processed food sales (top 10 are listed in Table 1) and local, small companies account for the other 80 % of the market share across the world. This decentralization poses a challenge for a concerted, global effort in translational research to ensure and improve nutrient security and sustainability worldwide and in view of a growing population.

**Table 1:** Percentage of manufacturers’ global packaged food retail sales

Top 10 companies	Region					
	Western Europe	Eastern Europe	North America	Latin America	Asia Pacific	World
Nestlé S.A.	2.9	2.6	3.9	6.0	1.8	3.3
Kraft Foods Inc.	1.9	1.8	7.0	1.7	0.7	2.6
Unilever Group	3.1	1.4	2.2	2.4	0.6	2.1
PepsiCo Inc.	0.9	0.7	4.6	3.1	0.3	1.8
Danone Groupe	1.9	1.4	0.7	1.4	0.7	1.3
Cadbury Schweppes Plc	1.4	0.7	0.7	1.5	0.4	1.0
Mars Inc.	1.2	0.9	1.9	0.2	0.2	1.0
Kellogg Co.	0.5	–	2.3	0.8	0.1	0.8
General Mills Inc.	0.2	–	2.5	0.2	0.2	0.7

*Source* Economic Research Service (ERS) of the United States Department of Agriculture (USDA) http://www.ers.usda.gov/topics/international-markets-trade/global-food-markets/global-food-industry.aspx (Accessed 16 Jan 2015). Original source: Euromonitor 2009

A long-term approach to assess sustainable nutrition security over the next 35 years has been initiated by ILSI’s Center for Integrated Modeling of Sustainable Agriculture and Nutrition Security (Acharya et al. 2014). Caloric and nutrient adequacy, dietary quality, dietary diversity, dietary sustainability, consumer choice, and the resiliency of the food system will be measured across space and time to provide quantitative assessments of nutrient security. These data-driven nutrient metrics will be integrated with measuring the social, environmental, and economic sustainability (Institute of Medicine 2015).

**Fig. 3:**
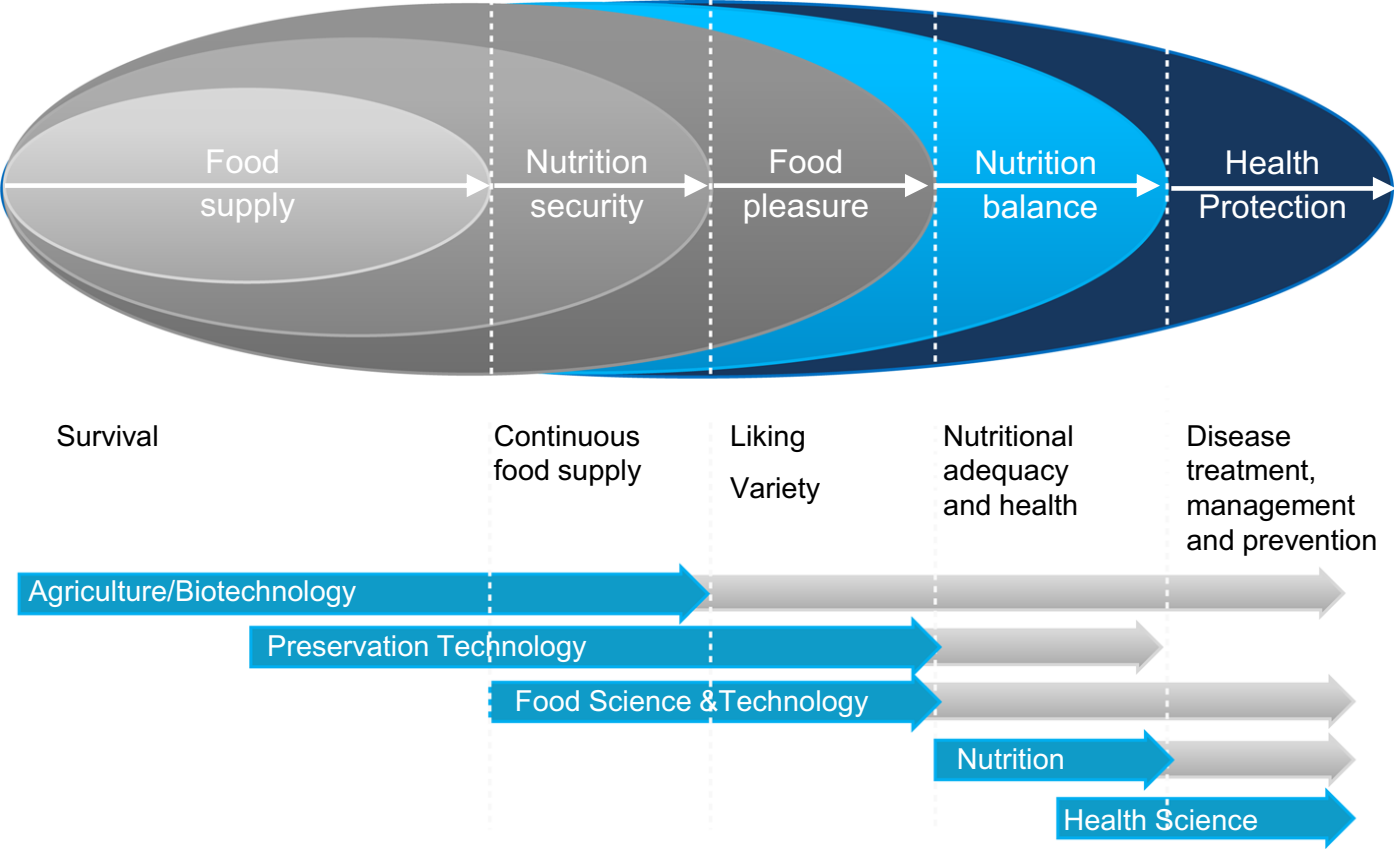
Nutrient security sciences. The nutrient chain extends from a secure food supply through health protection. Basic sciences in multiple disciplines play a role in this chain, but often are conducted independently without links of common language, methods, or results

Nutrition research plays a significant role in the nutrient chain from agriculture to food and health maintenance (Fig. 3). Such comprehensive, integrated systems research facilitates the understanding of: (i) the nutrient composition of foods; (ii) how nutrients can be preserved to deliver (fresh or processed) safe, nutritious, and affordable foods (Fig. 3); and (iii) how to optimize nutrient intake for sustaining health (Hammond and Dubé 2012; Acharya et al. 2014; Herforth et al. 2014). We discuss here how nutrient quality is influenced by crop genetics, agricultural environments, and potential losses between seed and fork. These and other factors have direct consequences for nutrition and biomedical research. We also present a key concept still missing in many discussions of nutrition sustainability: What is nutritional health and how is it measured?

## Agriculture and nutritional quality

Much of the thinking on food and nutrition sustainability emanated from a revaluation of the green revolution (GR) that dramatically increased grain and rice production (Pingali 2012). Enough food was grown during its initial phase (1966 through 1985) for an estimated 1 billion people. Yields for wheat, rice, maize, potato, and cassava in all developing countries increased by 208, 109, 157, 78, and 36 %, respectively (Pingali 2012). Although a success in terms of crop yield and benefits of saved lands converted to agriculture, the green revolution also produced unintended ecological consequences leading to a slowdown in yield growth: water overuse and waste, soil degradation, increased pesticide use, and chemical runoff are decreasing crop growth yields and raising justified concerns about the sustainability of the current agricultural methods (De Fraiture et al. 2010). Management of water is perhaps the most challenging of these factors since the agricultural sector consumes ~70 % of this resource that cannot be replenished. In contrast, agronomic biofortification with mineral fertilizers (e.g., zinc, nickel, iodine, copper) may improve nutritional quality of crops (La Frano et al. 2014) although repeated doses may be necessary due to weather, growth, and runoff. Non-discriminate use of single or a few nutrients—for example just nitrogen or nitrogen plus phosphorous in soils lacking calcium or magnesium—would not induce complete recovery of other lost nutrients: Liebig’s law of the minimum holds that optimal growth, development, and performance will be limited by the least available essential input (Sands et al. 2009). Crop growth (and indeed, human growth) is best optimized by balanced nutrient availability and water—a powerful illustration why it takes an integrated systems view for sustaining agriculture and nutrition.

The reliance on the best high-yield-variant (HYV) crops has led to an estimated 75 % loss in genetic diversity of agricultural plants and animals between 1900 and 2000 (FAO 2010). Over the last 10,000 years, humans may have used as many as 7000 different plants for food and other basic needs (Esquinas-Alcázar 2005). The majority of humans now cultivate about 150 plants for food, with 12 species supplying most of our nutrients (Esquinas-Alcázar 2005). An international awareness of the importance of the plant genetic diversity has led to the creation of about 1750 gene banks conserving about 7.4 million samples. The FAO spearheaded an international treaty for Plant Genetic Resources ratified by 125 of 193 nations (http://www.planttreaty.org/).

The loss of genetic diversity in agriculture not only limits potential nutritional diversity (Charrondière et al. 2013) but increases the vulnerability to sudden changes in climate and to the appearance of new pests and diseases. Plants with robust phenotypic flexibility are more likely to survive in the fluctuating local and regional environments (Gratani 2014) that are emerging as the planet’s atmosphere warms (Kimoto et al. 2013). Phenotypic plasticity is the change in phenotype expressed by a single genotype in different environments, a concept of resilience applicable to plants (Gratani 2014), humans (Van Ommen et al. 2009), and other organisms. Such flexibility allows the organism to function in diverse conditions—the ability to function in a given environment can be defined as an organism’s health status. However, a consequence of flexibility is that the same cultivar of a crop plant will have different nutrient contents when grown in local soils in climatically and geographically different environments (e.g., Di Silvestro et al. 2012; Charrondière et al. 2013). Similar concepts of resilience also apply to ecosystems (Edenhofer et al. 2014). The net result of modern agricultural practices is that fresh food, and food processed from it, may have inferior nutritive value compared to before the Green Revolution (Pearson 2009).

These variations in nutrient composition may be even further amplified during the transport of and processing of fresh foods. Nutrient-affecting post-harvest activities include handling, storage, processing, packaging, and transportation. Losses are often measured in weight (e.g., Parfitt et al. 2010) which may obscure losses of specific nutrients. Grains, tubers, fruits, and vegetables all have unique requirements to maintain nutritional quality post-harvest but share the sensitivities to loss of water, physiological deterioration, mechanical damage, diseases, and pests (FAO 1989). A few examples of reasons for post-harvest loss include (Gustavsson et al. 2011):

- Hygiene of water for irrigation and post-harvest washing: Contaminated water introduces pathogens such as *Bacillus cereus*, *E. col O157:H7*, *Salmonella spp*, and *Listeria monocytogenes* (Camelo 2004).
- Type of product: Cereals have lower losses per ton than vegetables and fruits (Parfitt et al. 2010). Estimates vary widely, but rice is an example: Only ~5 % of rice is lost in India but up to 80 % may be lost in extreme conditions in Vietnam (Parfitt et al. 2010).
- Genotype of product: Carotenoid loss varied under identical processing and storage conditions in different lines (isolates of breeding crosses) of high-carotenoid maize (Burt et al. 2010). These differences may be due to genetic differences in carotenoid synthesis and degradation.
- Distance to market: Primary production to market is shorter in rural areas in low- and middle-income countries (LMIC) and longer in high-income countries or urban areas in all economies.
- Storage facilities and transport conditions: Rural homes have less sophisticated storage systems and higher losses. Certain crops, such as tomatoes (Passam et al. 2007), are more sensitive to cold (or heat) than other crops.
- Food processing and cooking: These are methods that directly or indirectly cause loss of nutrients. Air drying *versus* high-temperature drying alters loss of carotenoid in maize (Burt et al. 2010), and provitamin A precursors in fortified rice are differentially affected by conditions of boiling or frying (Wieringa et al. 2014).
- High-income countries may lose an estimated 33 % of food after purchase by consumers.

### Why these facts matter for human research

Measuring nutrient loss across all post-harvest conditions is impractical and expensive. Yet, human research studies require knowledge of habitual or daily food consumption (Tucker 2007; Stumbo et al. 2010; Tucker et al. 2013) since physiology adapts to nutrient intakes. Food frequency questionnaires, 24-h diet recalls, and food diaries are typically used to assess habitual (FFQs) or daily food intake (recalls and diaries) (Observatory 2010). These food records are converted to nutrient information using food composition databases such as the USDA (http://ndb.nal.usda.gov/) or European databases (Finglas et al. 2014). The USDA database reports a single average value with standard error (*n* ≤ 6) for the nutrient measured, and other databases provide ranges for individual chemicals (e.g., Foodb database (http://foodb.ca/)). Most of these databases aggregate information from multiple analytical reports conducted over decades on cultivars growing in specific environments (Pennington et al. 2007; McCabe-Sellers et al. 2008b; Charrondière et al. 2013). While nutrient intake measurements can never be biochemically accurate because of the (i) farm-to-fork losses in nutritional quality and (ii) recall bias of human subjects (Kristal et al. 2005), knowing the food groups and the approximate range of nutrient intake is nonetheless necessary for health research since phenotypes result from gene–environment interactions and food is the most important factor to maintain life.

## Genomics and agricultural nutritional quality

Delivering the right nutrients to populations and individuals is challenging given the inconsistencies in ensuring nutritional quality across the nutrient chain. Humans began selecting crops through breeding at the dawn of the agriculture (~10,000 years ago) while the pre-genomic modern era of plant genetics emerged in the early 1900s based on the work of Mendel, Correns, de Vries, and von Tchsermak (Acquaah 2007). Classic plant breeding technology requires 10–15 years to select a new hybrid and bring it to market (Duvick 1986). However, newer methods such as somatic embryogenesis or marker-assisted selection (MAS) are used in order to speed up this selection process (Fehér 2014).

### Genetic modifications to increase yield

 The first commercialized, genetically modified (GM) plant was soybean containing the 5-enolpyruvylshikimate-3-phosphate synthase (EPSPS) gene from *Agrobacterium tumafaciens* (EFSA GMO Panel Working Group on Animal Feeding Trials 2008). This variant of EPSPS is resistant to glyphosate, a widely used herbicide developed by Monsanto. The EPSPS reaction is a key step in producing the aromatic amino acids phenylalanine (Phe), tryptophan (Trp), and tyrosine (Tyr). Phe and Trp are essential amino acids in humans because humans lack the EPSPS reaction or other aromatic amino acid biosynthetic pathways. Hence, glyphosate would only affect humans through off-target effects, although none have been reported.

The mode of action of insecticides is more complex since the plant first synthesizes a bacterial protein (example: Cry proteins, typically from *Bacillus thuringiensis*), which—when ingested and degraded by an insect—produces a toxin that binds specifically to a cell adhesion receptor in the insect midgut (Hernández-Rodríguez et al. 2013). The use of these bacterial proteins as natural insecticides is common in organic cultivation and has a long history of safe use. GM crops have been shown to be safe in animal studies (EFSA GMO Panel Working Group on Animal Feeding Trials 2008; Domingo and Giné Bordonaba 2011) and in humans (Committee on Identifying and Assessing Unintended Effects of Genetically Engineered Foods on Human Health 2004; König et al. 2004; EFSA GMO Panel Working Group on Animal Feeding Trials 2008; Domingo and Giné Bordonaba 2011; Ammann 2014). Transgenic proteins used as insecticides are degraded similarly to native proteins due to food production methods and digestion in vivo (Hammond and Jez 2011; Hammond et al. 2013). In addition, the nutritional content of GM crops has repeatedly been shown to be substantially equivalent to non-transformed crops, when cultivar and environmental variations are taken into consideration (Catchpole et al. 2005; Shewry et al. 2007; Bøhn et al. 2014).

GM crops have been planted on over 160 million hectares by 2012 by 17.2 million farmers (Brookes and Barfoot 2014). The high rate of adoption by farmers is driven by the economic benefits associated with GMO cultivation, mainly due to reduced use of pesticides and labor. Insecticide use has decreased with adoption of insect-resistant GMOs, and herbicides-tolerant crops have enabled the use of glyphosate which replaced more toxic and persistent herbicides (Fernandez-cornejo et al. 2014). Nevertheless, due to over reliance on glyphosate, resistance has developed in some weed species.

### Biofortification

Another emerging approach that is showing promising results to address micronutrient deficiencies is the development and dissemination of biofortified crops like sweet potato and cassava. Conventional breeding techniques are used in combination with genomic technologies to select plant varieties for variants with naturally higher content of provitamin A. Biofortification could also be accomplished by methods such as introducing foreign DNA, through selective breeding, or using newer genomic technologies (Bouis et al. 2011; Chen and Lin 2013; De Moura et al. 2013), but these technologies may not always shorten the development time from concept to commercialized crop (Bouis et al. 2011). A partial list of crops modified to date includes (from Chen and Lin 2013):

- Rice with β-carotene (0–37 μg/g), iron (increased >sixfold), or folate (<1–17 μg/g)
- Maize with ascorbate (about fivefold increase)
- Soybean with oleic acid (fourfold increase)
- Canola with omega-3 fatty acid (fourfold increase)
- Wheat with amylose (threefold increase)
- Tomato with anthocyanin (0–2.83 μg/g).

Harvest Plus, an organization that drives biofortification worldwide as part of the Consultative Group on International Agricultural Research (CGIAR) Program on Agriculture for Nutrition and Health (A4NH), lists seven biofortified crops that were developed with conventional breeding technologies combined with genomic selection (Table 2 and described below).

**Table 2:** Biofortified crops

Target crop	Nutrients	Introduced to	Release dates
Bean	Iron	DR Congo, Rwanda	2012
Cassava	Vitamin A	DR Congo, Nigeria	2011
Maize	Vitamin A	Nigeria, Zambia	2012
Pearl millet	Iron	India	2012
Rice	Zinc	Bangladesh, India	2013
Sweet potato	Vitamin A	Mozambique, Uganda	2007
Wheat	Zinc	India, Pakistan	2013

*Source* http://www.harvestplus.org/content/crops

Golden rice, which was developed using genetic modification, is the “poster child” for a nutritionally enhanced crop. The first generation of golden rice introduced the entire β-carotene pathway via two vectors using an Agrobacterium-mediated transformation (Ye et al. 2000). One vector encoded phytoene synthase (*psy*) with a nearby transit peptide (*tp*) gene from daffodil (*Narcissus pseudonarcissus*) under the control of the endosperm-specific glutelin (Gt1) promoter and bacterial (*Erwinia uredovora*) phytoene desaturase (*ctrl*) and *tp* gene controlled by the constitutive cauliflower mosaic virus promoter (CaMV 35S). A second vector contained *Narsicissus pseudonarcissus* lycopene β-cyclase (*lcy*) controlled by the rice Gt promoter and the *aphIV* gene controlled by CaMV 35S. The first-generation rice crop produced about 1.6 μg/g, but yield has been improved almost tenfold by altering the source of the *psy* gene (Paine et al. 2005). However, to date, golden rice has not been fully commercialized due to the controversies surrounding the use of GM technologies. The economic cost of this delay to India alone has been calculated to be USD$199 million annually since 2002 (the original expected year of introduction). The disability-adjusted life year (DALY) of vitamin A deficiency that resulted was estimated to be 1,424,680 life years over a 10-year period for India alone (Wesseler and Zilberman 2014).

The majority, if not all, genetic modifications have been achieved through the introduction of one to several genes (Chen and Lin 2013) with some of those genes being derived from bacteria. Concerns about introducing foreign DNA across species (Ronteltap et al. 2007; Chassy 2010) have generated intense debate and stringent regulatory oversight (Dong et al. 2008; Ammann 2014; Devos et al. 2014). However, direct modifications to plant genomes may be done with new genomic technologies (described below) without the need to introduce foreign DNA (Lusser et al. 2012) and, hence, may render introduction of foreign DNA into plants obsolete and outside of the regulatory oversight for genetically modified organisms (Pauwels et al. 2014).

Existing technologies for direct modifications of plant genomes include cytogenetic mapping (Figueroa and Bass 2010) to more precisely identify genes of interest. Genomic methods such as marker-assisted selection [MAS or marker-assisted breeding—(Miedaner and Korzun 2012)], marker-assisted recurrent selection [MARS (Bohra 2013)], and genomic selection [GS (Jannink et al. 2010)] allow more rapid selection of phenotypic traits. The advantage of these methods is that natural varieties are selected and they are not genetically modified. Hence, these selection methods avoid the controversy of GM plants and have no regulatory hurdles. These methods, however, are being superseded by the technological ability to edit genomic sequences at defined sites (Doudna and Charpentier 2014), which is emerging as a transformative technology for plant breeding (Baltes and Voytas 2014). Genome editing relies on the use of site-specific nucleases to introduce double-strand breaks that are repaired by host factors involved in homologous recombination (HR) or non-homologous end joining recombination (NHEJ). The double-strand breaks can be targeted by engineered hybrid proteins consisting of zinc finger-binding domains linked to the nuclease domain of *FokI* creating zinc finger nucleases [ZFN (Jabalameli et al. 2014)]. TALENs is an acronym for transcription activator-like effector nucleases which, like the zinc nucleases, is an engineered hybrid containing the DNA-binding domain of transcription activator-like effectors (TALEs) linked to double-stranded nuclease (Kim and Kim 2014). CRISPr technology (clustered regularly spaced palindromic repeat) emerged from research on adaptive anti-viral immunity in bacteria (Doudna and Charpentier 2014). CRIPRr differs from other genome-editing technologies in that it relies on RNA to activate and target the CRISPr-associated protein 9 (Cas9). Since the single-chain guide RNA (sgRNA) sequences can be easily engineered into the CRISPr vector, no complicated protein engineering is necessary. Hence, targeting specific sequences to modify within genomes has become relatively straightforward.

However, none of these methods produce predictable results unless the trait depends on a single genetic locus: Which gene or how many genes would have to be modified to alter a phenotype—improve the levels of a nutrient? Advances in computational analysis (reviewed in Bohra 2013) are aimed at identifying many (at least 80) quantitative trait loci (QTL) responsible for some complex phenotypes. Next-generation sequencing of whole genomes will likely further speed developments in plant genomics (Varshney et al. 2014). Combined with MAS (which allow the transfer of phenotypes that depend on the action of several genes), this approach is seen as holding more promise for selecting plant phenotypes. Nevertheless, the key limitation to improve nutritional quality of plants using these new technologies will be how to identify and control the many loci involved in complex traits.

In addition to the technologies, the bioavailability of micronutrients in biofortified materials may also vary considerably. Provitamin A (beta carotene) is very well absorbed from biofortified roots, tubers, and cereals, but the presence of phytates and other antinutrients in grain crops—whether biofortified or not—hinders the absorption of minerals such as iron and zinc (La Frano et al. 2014). However, since a higher total amount of mineral micronutrients is present in biofortified crops, the final amount absorbed is also higher. Total iron absorption by young women from iron-biofortified pearl millet composite meals is double than that from regular millet meals but less than that from post-harvest iron-fortified millet meals (Cercamondi et al. 2013). Through existing methods such as the Mexican traditional nixtamalization (Tovar and Larios-Saldaña 2005) or other modern industrial technologies, food processing has a good potential to neutralize or modify the antinutrients to improve micronutrient absorption (Luo et al. 2010).

## Food processing

The complexity of improving agricultural products at the source, through classical or marker-assisted breeding, or through genome technologies may delay the improvement of nutritional quality. While large-scale food processing and manufacturing by the food industry is at times viewed negatively, processing agricultural products produces safe, shelf-stable, and nutritious foods that are an integral component of healthy diets for the world’s populations (Dwyer et al. 2012). Processed foods range from minimally processed (e.g., coffee beans) to complex meals (e.g., frozen dinners). Food processing by humans has occurred since the control of fire with archeological evidence of food manipulation ranging from 250,000 to 800,000 years ago (Armelagos 2014). The transition from foraging to farming about 10,000 years ago spurred innovations in food processing and storage, as exemplified by breads, cheeses, wine, and beer. Ironically, this initial foray into food processing caused a decrease in nutrient quality for our agrarian ancestors (Armelagos 2014).

The ability to extract chemicals from agricultural plants and farm animals was developed in the modern era of industrialization beginning in the mid-1800s and enabled the creation of new food combinations. In many cases, the driving forces for food processing were (and still are) enhanced safety, prolonged shelf life, and lowered cost but also reduced post-harvest losses. Besides these economic and risk management reasons, food production was also driven by aspects of pleasure and health, such as convenience, greater choice, and dietary diversity (Dwyer et al. 2012). However, concern is growing about the sustainability of current consumption and production patterns as well as the implications for nutritional outcomes resulting from the enhanced availability and affordability of food. The food processing sector is increasing efforts to improve efficiencies, reduce waste and losses along the supply chain and to raise the nutritional content of foods. In addition to providing calories and macronutrients (i.e., bulk carbohydrate, fat, and protein), the ability to fortify foods with micronutrients (i.e., vitamins, essential fatty/amino acids, minerals) has dramatically improved personal and public health for more than 70 years, at least in high-income countries (Dwyer et al. 2012; Semba 2012). Processed foods are becoming an increasingly important contributor to the nutrient chain since about ~75 % of the 10 billion world population in 2050 are expected to live in large urban areas, a population transition that is already altering sustainable ecosystems, goods and services (Herforth et al. 2014; Cumming et al. 2014).

## Improving nutrition for humans

The scientific and translational progress for producing more food and better-quality nutrition that has occurred or is emerging (Kim et al. 2013; Acharya et al. 2014; Lachat et al. 2014; Herforth et al. 2014; Allen et al. 2014) relies on one critical unknown: How does one define health for individuals? That is, what is the “ideal” nutritional content of a crop or food? Type 2 diabetes is an example of the complexity of physiology (in this case, a disease): The clinical markers of this disease can be caused by alterations in many different, independent molecular pathways (Kaput et al. 2007; Kussmann et al. 2013). Health also widely varies between individuals, and this interindividuality can be explained by the complex interactions within a genome and between the genome and the environment. Interindividuality was discussed in the modern era in *Biochemical Individuality* (Williams 1956), a book published about 3 years after the discovery of the structure of DNA.

**Fig. 4:**
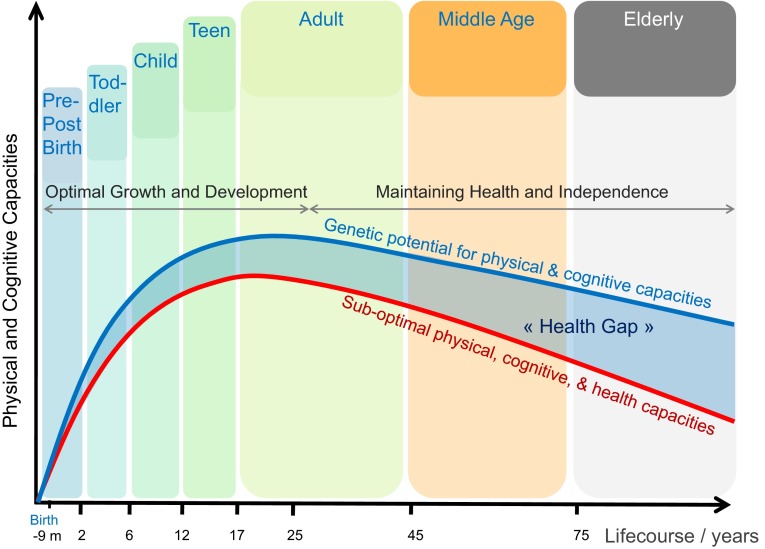
Life course and health gap. The life course is influenced by nutrition and other lifestyle and socioeconomic factors. A gap may exist between suboptimal capacities and the genetic potential. Closing this gap is a major focus of improving the nutrient chain for individuals in world populations. Source: http://www.nestle.com/asset-library/documents/creating-shared-value/nestle-csv-full-report-2014-en.pdf

In spite of over a century of intense biomedical research, the current knowledge is still insufficient in detail to guide recommendations, set agricultural programs, or plan for the needs of future generations. Hence, assessing which nutrients and how much of those nutrients are needed for growth, development, and maintenance of health of individuals during the life course (Fig. 4) are the defining questions for nutritional research now and in the future (Kaput and Morine 2012). Several factors contribute to this persisting lack of knowledge:

1. Although human physiology, environment, and genetic diversity and their interactions are highly complex, nutrition and biomedical research developed and pursued reductionist strategies that sought to describe the role of a single factor, such as a gene or a nutrient, on biological processes. Systems-level thinking and approaches are now seen as essential approaches for health research (e.g., (Panagiotou and Nielsen 2009; van der Greef et al. 2010; Afacan et al. 2012; Civelek and Lusis 2013; Kaput et al. 2014). Such systems are reflected by inter-nutrient, inter-gene, and gene–nutrient interactions, rather than by studying nutrients or genes in isolation. An added complication may be trade-offs that may occur when different health or disease outcomes are possible (heart health might require different levels of nutrients than gastrointestinal health).
2. Biomedical research focused primarily on European or their genetic descendants for reasons of convenience. Hence, a critical lack of data and knowledge exists for the nutrient requirements for individuals and populations living in diverse cultural and environmental conditions (Kaput et al. 2014).
3. Biomedical and nutritional research studies are usually limited in scope. Cost, lack of expertise, and logistic complexity contributes to the lack of sufficient details about diets and local environmental conditions resulting in limited ability to compare results from different studies. Nutrient uptake and utilization are influenced by conditions in the built environment, not only access to clean water and sanitation (Fig. 2), but also personal, socioeconomic, and political conditions. Analyzing this complex matrix of nutrient ratios, built environments, and socioeconomic variations and their effect on health would not be possible because of the sheer number and diversity of conditions. However, an emerging concept in ecological research called “the ecology of place” may provide a road map for nutrition research (Price and Billick 2010). The ecology of place or place-based research not only gathers data from local ecosystems but also uses a more complete description of the environmental context in design and interpretation of the results. Key goals and outcomes of this research strategy are to extract “portable ecological knowledge” from the small sample of systems that can be generalized or used to understand distinct ecologies of other places. Similar conceptual thinking may be applied to the diverse nutritional and genetic combinations occurring across the world: Well-designed nutritional research strategies produce data and results that can inform and scale to other populations. For example, translational research that combines principles of community-based participatory research with physiological (e.g., omics) assessments provides scientific knowledge that can be used across populations (McCabe-Sellers et al. 2008a; Monteiro et al. 2014; Morine et al. 2014) and creates shared value for participants and researchers.
4. Determining phenotypic plasticity or flexibility has been mentioned in regard to plants (Gratani 2014) but also applies to humans (Van Ommen et al. 2009, 2014). Phenotypic flexibility is exemplified by the oral glucose tolerance test: The metabolism of a (usually) 75-g dose of glucose over 1–2 h provides an assessment of an individual’s ability to metabolize simple carbohydrates. The link to long-term health is exemplified by the fivefold increase in risk for type 2 diabetes mellitus (T2DM) for women who have gestational diabetes (Buchanan et al. 1998; Gunderson et al. 2011; Konig and Shuldiner 2012). Omics technologies have greatly expanded the information that can be attained from oral glucose tolerance tests [OGTT—(Morris et al. 2013; Ho et al. 2013)]. The challenge concept has been extended to oral lipid and mixed meal tests [rev in (Stroeve et al. 2015)]. The acute challenge concept can be extended to short-term interventions with high but safe doses of bioactives that are typically present in foods at low concentration and require a longtime to produce phenotypic changes (Monteiro et al. in preparation). While such nutritional studies sound unique, the underlying concept asks how individuals partition nutrients post-prandially, and how differences in those processes determine long-term health.

## Conclusions

Regardless of the approaches and modeling that are planned, data from well-defined human research studies that account for genetic and environmental influences on individuals will be necessary to understand how to optimize the complex nutrient chain. Biomedical research data will contribute to the systems approaches to nutrition security and sustainability that are being developed across multiple sectors (Wahlqvist and Kuo 2009; Acharya et al. 2014; Vieweger and Döring 2014; Herforth et al. 2014; Allen et al. 2014). While this rethinking of the conceptual framework of modern biomedical research (Kaput et al. 2014) will be a challenge to scientists and to the “small” team approach of current nutrition research, adapting a multi-disciplinary systems approach will be necessary to improve the health of individuals and populations now and in the future.
